# Adiposity and lipid metabolism indicators mediate the adverse effect of glucose metabolism indicators on oogenesis and embryogenesis in PCOS women undergoing IVF/ICSI cycles

**DOI:** 10.1186/s40001-023-01174-8

**Published:** 2023-07-03

**Authors:** Huahua Jiang, Manfei Si, Tian Tian, Huifeng Shi, Ning Huang, Hongbin Chi, Rui Yang, Xiaoyu Long, Jie Qiao

**Affiliations:** 1 Center for Reproductive Medicine, Department of Obstetrics and Gynecology, Peking University Third Hospital, Beijing, 100191 China; 2 National Clinical Research Center for Obstetrics and Gynecology, Peking University Third Hospital, Beijing, China; 3 Key Laboratory of Assisted Reproduction, Peking University, Ministry of Education, Beijing, China; 4 Beijing Key Laboratory of Reproductive Endocrinology and Assisted Reproductive Technology, Beijing, China; 5 Department of Obstetrics and Gynecology, Peking University Third Hospital, Beijing, China; 6 National Centre for Healthcare Quality Management in Obstetrics, Beijing, China; 7 Beijing Advanced Innovation Center for Genomics, Peking University, Beijing, China; 8 Peking-Tsinghua Center for Life Sciences, Peking University, Beijing, China

**Keywords:** PCOS, Mediating effect, Adiposity and lipid metabolism, Glucose metabolism indicators, IVF/ICSI outcomes

## Abstract

**Background:**

Polycystic ovary syndrome (PCOS) women have high incidences of dyslipidemia, obesity, impaired glucose tolerance (IGT), diabetes, and insulin resistance (IR) and are fragile to female infertility. Obesity and dyslipidemia may be the intermediate biological mechanism for the associations between glucose metabolism dysfunction and abnormal oogenesis and embryogenesis.

**Methods:**

This retrospective cohort study was performed at a university-affiliated reproductive center. A total of 917 PCOS women aged between 20 and 45 undergoing their first IVF/ICSI embryo transfer cycles from January 2018 to December 2020 were involved. Associations between glucose metabolism indicators, adiposity and lipid metabolism indicators, and IVF/ICSI outcomes were explored using multivariable generalized linear models. Mediation analyses were further performed to examine the potential mediation role of adiposity and lipid metabolism indicators.

**Results:**

Significant dose-dependent relationships were found between glucose metabolism indicators and IVF/ICSI early reproductive outcomes and between glucose metabolism indicators and adiposity and lipid metabolism indicators (all *P* < 0.05). Also, we found significant dose-dependent relationships between adiposity and lipid metabolism indicators and IVF/ICSI early reproductive outcomes (all *P* < 0.05). The mediation analysis indicated that elevated FPG, 2hPG, FPI, 2hPI, HbA1c, and HOMA2-IR were significantly associated with decreased retrieved oocyte count, MII oocyte count, normally fertilized zygote count, normally cleaved embryo count, high-quality embryo count, or blastocyst formation count after controlling for adiposity and lipid metabolism indicators. Serum TG mediated 6.0–31.0% of the associations; serum TC mediated 6.1–10.8% of the associations; serum HDL-C mediated 9.4–43.6% of the associations; serum LDL-C mediated 4.2–18.2% of the associations; and BMI mediated 26.7–97.7% of the associations.

**Conclusions:**

Adiposity and lipid metabolism indicators (i.e., serum TG, serum TC, serum HDL-C, serum LDL-C, and BMI) are significant mediators of the effect of glucose metabolism indicators on IVF/ICSI early reproductive outcomes in PCOS women, indicating the importance of preconception glucose and lipid management and the dynamic equilibrium of glucose and lipid metabolism in PCOS women.

**Supplementary Information:**

The online version contains supplementary material available at 10.1186/s40001-023-01174-8.

## Background

Polycystic ovary syndrome (PCOS), characterized by oligo-anovulation, hyperandrogenism, and polycystic ovarian morphology, is the most common endocrine disease affecting 4 to 21% of reproductive-aged women [1–3]. PCOS has diverse lifelong clinical manifestations of reproductive, metabolic, and psychological disorders, such as anovulatory infertility, dyslipidemia, obesity, impaired glucose tolerance (IGT), Type 2 diabetes mellitus (T2DM), insulin resistance (IR), cardiovascular disease, low self-esteem, psychosexual dysfunction, depression, and anxiety [1, 2, 4, 5]. PCOS women undergoing adverse assisted reproductive technology (ART) are at high risk of adverse IVF outcomes [6]. The pathogenesis of PCOS is complex, including genetic and epigenetic susceptibility, hypothalamic and ovarian dysfunction, hyperandrogenism, hyperinsulinemia, and adiposity-related mechanisms [2, 7].

As common manifestations of PCOS, maternal lipid and glucose metabolism dysregulations have been suggested to contribute to abnormal embryonic development [8–12]. In vitro fertilization (IVF)-based studies indicated that poor embryogenesis and pregnancy outcomes in diabetic and obese mice result from the maternal effects on the oocytes rather than the uterine environment, suggesting an oocyte origin of adverse reproductive outcomes [11, 13]. IVF/intracytoplasmic sperm injection (ICSI) provides an excellent means to study the effects of maternal factors on oogenesis and embryogenesis. The recent clinical studies have shown that dyslipidemia, obesity, glucose homeostasis dysregulation, IR, and metabolic syndrome (MetS) contribute to ART outcomes in PCOS women. A multicenter randomized controlled trial of 1508 PCOS women observed that MetS affects female fecundity in forms of longer infertility duration, poorer ovarian response, fewer retrieved oocytes, fewer available embryos, and lower chance of cumulative live birth [14]. Several studies have also reported the impact of sole obesity and IR on pregnancy outcomes in PCOS women undergoing IVF/ICSI [15–17]. However, these results remain controversial [18–20], and it is not easy to explore causal paths due to the close interactions among metabolite profiles.

The causal roles of body mass index (BMI) and IR in PCOS etiology have been proven via Mendelian randomization (MR) analyses [21–23]. However, the causal relationship between glucose and lipid metabolism in PCOS women remains uncertain. A small MR analysis in the Netherlands indicated that dyslipidemia (i.e., high plasma triglycerides (TGs) levels) seem to be responsible for hyperglycemia and T2DM risk, and large (L) and extra-large (XL) high-density lipoprotein (HDL) lipid components might protect against increasing fasting glucose level [24]. However, a recent MR study based on the genome-wide association summary statistics of Europeans reported bidirectional causality among serum glycemic and lipid traits [25]. Therefore, we assume the potential mediation role of adiposity and lipid metabolism indicators on the associations between glucose metabolism indicators and IVF/ICSI outcomes in PCOS women.

When compared with MR analysis, mediation analysis is another valuable tool for exploring causality by testing whether a variable is a mediator and what proportion of the role it plays [26]. In this study, we aimed to determine whether adiposity and lipid metabolism indicators mediate the effect of glucose metabolism indicators on early reproductive and pregnancy outcomes in PCOS women undergoing IVF/ICSI cycles.

## Materials and methods

### Ethical approval

This study was approved by the Institutional Review Board of Peking University Third Hospital (No. 2021SZ—011). All the patients had previously provided written informed consent.

### Study population

**Fig. 1:**
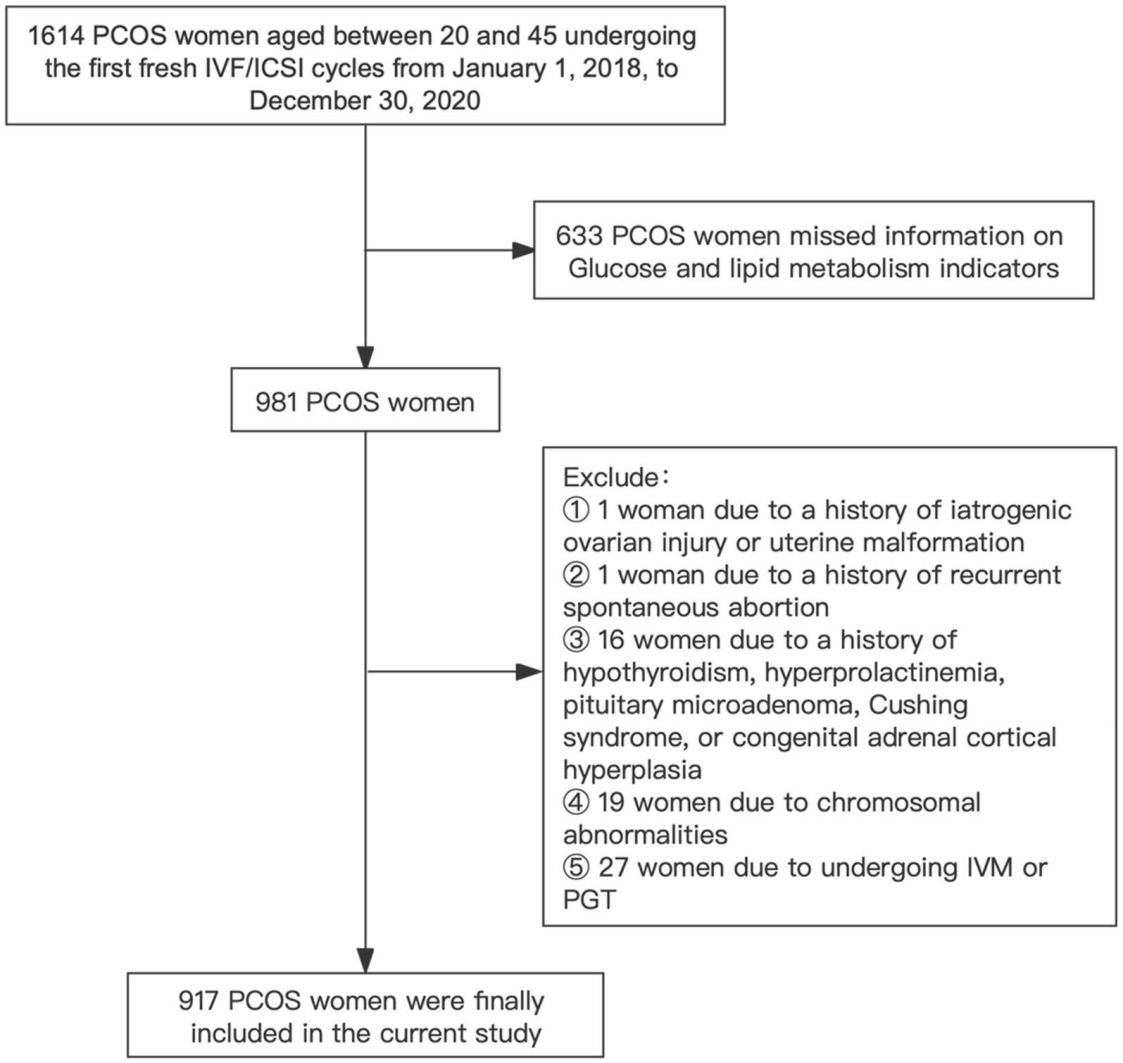
Flow chart for the study population to investigate associations between sleep characteristics and outcomes of IVF/ICSI

We performed a retrospective cohort study of 917 PCOS patients aged between 20 and 45 undergoing the first fresh IVF/ICSI cycles from January 1, 2018, to December 30, 2020, at the Reproductive Center of Peking University Third Hospital (Fig. 1). Diagnosis of PCOS is based on the 2003 Rotterdam criteria and confirmed with at least two of the following three features: hyperandrogenism (clinical or biochemical), menstrual abnormalities (oligo- or amenorrhea), and polycystic ovary morphology (≥ 12 antral follicles (2 to 9 mm in diameter) in unilateral or bilateral ovaries or ovarian volume > 10 cm^3^ on ultrasonography) [27]. The exclusion criteria were as follows: (1) history of chemotherapy, radiotherapy, ovarian tumors, or ovarian surgery; (2) uterine malformation; (3) history of recurrent spontaneous abortion or autoimmune disease; (4) history of hypothyroidism, hyperprolactinemia, pituitary microadenoma, Cushing syndrome, or congenital adrenal cortical hyperplasia; (5) chromosomal abnormalities; (6) in vitro maturation or preimplantation genetic testing cycles. The information on baseline reproductive and cycle characteristics were collected from the internal electronic medical records. The information on pregnancy outcomes (i.e., biochemical pregnancy, clinical pregnancy, and live birth) was collected by experienced follow-up nurses using telephone interviews.

### Standardized IVF procedures

We followed the standardized protocols of ovarian stimulation procedures, oocyte retrieval, insemination, embryo transfer, and luteal support as previously described [28, 29]. The initial dose of gonadotropins for ovarian stimulation was individualized based on the BMI and ovarian reserve features. The insemination technique (i.e., conventional insemination, ICSI) was applied based on the sperm quality. The embryos were morphologically graded on Day 3 and Day 5.

### Definition of study outcomes

Normal fertilization is cognized as zygotes with two pronuclei (2PN). High-quality embryos are defined as 5 to 8-cell embryos with uniform size and less than 30% fragments. Biochemical pregnancy is defined as the positive serum β-hCG level 14 days after ET. Clinical pregnancy is confirmed by ultrasonographic visualization of one or more gestational sacs with fetal heart activity 28 days after ET. Live birth is the delivery of a live-born fetus beyond 28 weeks of gestation. Deliveries of multiple fetuses were counted as just one live birth.

### Laboratory assessment

Glucose and lipid metabolism indicators were measured within 6 and 3 months before ovarian stimulation, respectively. Fasting plasma glucose (FPG), fasting plasma insulin (FPI), 2-h plasma glucose (2hPG), and 2-h plasma insulin (2hPI) were obtained through a 75 g oral glucose tolerance test (OGTT) and insulin-releasing test after an overnight (at least 8 h) fast. FPG and 2hPG were measured by the hexokinase method (Beckman Access Health Company, Chaska, MN, USA); and FPI and 2hPI were measured by the chemiluminescence method using the Immulite 1000 system (DPC, USA). Glycosylated hemoglobin type A1C (HbA1c) was measured by ion-exchange high-performance liquid chromatography (HLC-723 G8, Tosoh, Japan). The serum total cholesterol (TC), TG, HDL cholesterol (HDL-C), and low-density lipoprotein cholesterol (LDL-C) were determined using a fully automatic biochemical analyzer (7170A, HITACHI, Japan). The intra- and inter-assay variations of all the glucose and lipid metabolism indicators were less than 10%. The technicians in the biochemical laboratory were blinded to other clinical information about the study population.

### Statistical analysis

All the statistics, calculations, and plotting were performed using the R software (version 4.0.3). For patients’ baseline reproductive and cycle characteristics, continuous data were presented as median (interquartile range (IQR)), and discrete variables were described as frequencies and percentages (*n* (%)). We computed the homeostasis model assessment 2 estimate of insulin resistance (HOMA2-IR) using the HOMA2 Calculator (version 2.2.4) (www.dtu.ox.ac.uk/homacalculator/index.php, University of Oxford) based on FPG and FPI values [30], and further divided the distribution of HOMA2-IR values into four groups according to quartiles (0.9, 1.3, and 1.9). We analyzed the baseline reproductive and cycle characteristics according to the HOMA2-IR quartile groups by applying Kruskal–Wallis tests for continuous variables and Chi-squared tests (or Fisher’s exact test) for categorical variables. Owing to right skewness, FPG, 2hPG, FPI, 2hPI, HbA1c, HOMA2-IR, TC, TG, HDL-C, LDL-C, and BMI were transformed by natural logarithm (ln) when analyses required a normal distribution.

We constructed multivariate generalized linear models (GLMs) to evaluate the associations between glucose metabolism indicators, adiposity and lipid metabolism indicators, and IVF/ICSI outcomes in PCOS women to assess the feasibility of further mediation analyses. A Poisson distribution and log link function were specified for retrieved oocyte count, MII oocyte count, normally fertilized zygote count, normally cleaved embryo count, high-quality embryo count, and blastocyst formation count, and a binomial distribution and logit link function were specified for biochemical pregnancy, clinical pregnancy, and live birth. According to the statistical consideration and previously reported biological plausibility [31, 32], the following variables were examined as potential covariates and potential confounders: age (continuous), basal follicle-stimulating hormone (FSH) (continuous), antral follicle count (AFC) (continuous), duration of infertility (continuous), infertility type (primary versus secondary infertility), ovarian stimulation regimen (GnRH antagonist protocol, long GnRHa protocol, others), insemination technique (IVF versus ICSI), number of transferred embryos (one vs. two), and timing of embryo transfer (Day 3, Day 5, Day 6). These abovementioned variables were retained in the final multivariate analyses if they caused a > 10% change in the effect estimates based on the “Change-in-Estimate Method” [33].

We further performed mediation analyses to evaluate whether adiposity and lipid metabolism indicators are potential mediators for the associations between glucose metabolism indicators (independent variables) and IVF outcomes (dependent variable) in PCOS women using the mediation package in R. Assuming the mediation analysis hold, we estimated the direct effect which refers to the effect of glucose metabolism indicators on IVF/ICSI outcomes in PCOS women when controlling for adiposity and lipid metabolism indicators. We also estimated the indirect effect that represents the mediating effect of adiposity and lipid metabolism indicators. The proportion of the mediation by each adiposity and lipid metabolism indicator was computed as the ratio of the indirect effect to the total effect. All statistical significance levels were set at two-sided *P* < 0.05.

## Results

### Participants’ characteristics

Table 1 presents the baseline reproductive and cycle characteristics of the 917 PCOS women. They had a median (IQR) age of 30.0 (28.0, 33.0) years and a median (IQR) BMI of 25.6 (22.6, 28.6) kg/m^2^, were predominantly diagnosed with primary infertility (71.3%), and 83.1% underwent GnRH antagonist protocol. When compared with women in the lowest quartile of HOMA2-IR, women in the highest quartile were more likely to have a longer duration of infertility (*P* = 0.02); higher FPG, 2hPG, FPI, 2hPI, HbA1c, BMI, TG, and LDL-C (All *P* < 0.001); and a lower HDL-C (*P* < 0.001). Women in the highest quartile of HOMA2-IR were more likely to undergo the conventional IVF procedure (77.8% vs. 66.1%; *P* = 0.02) and Day 3 embryo transfer (96.8% vs. 95%; *P* = 0.03) when compared with women in the lowest quartile. No other characteristics differed significantly across quartiles of HOMA2-IR.

**Table 1:** Baseline reproductive and cycle characteristics of the PCOS women undergoing the first fresh IVF/ICSI cycles

Characteristics	Overall *n* = 917	HOMA2-IR				*P*
		← More insulin resistant		Less insulin resistant→		
		0–0.9	> 0.9–1.3	> 1.3–1.9	> 1.9	
		*n* = 254	*n* = 213	*n* = 234	*n* = 216	
Age, years	30.0 (28.0, 33.0)	31.0 (29.0, 33.0)	31.0 (28.0, 33.0)	30.0 (28.0, 33.0)	30.0 (28.0, 33.0)	0.35
BMI, kg/m^2^	25.6 (22.6, 28.6)	22.3 (20.7, 24.5)	24.9 (22.3, 27.3)	27.2 (24.5, 29.5)	28.3 (25.9, 31.1)	< 0.001
AFC, *n*	17 (13, 22)	17 (13, 22)	17 (12, 22)	17 (12, 22)	17 (13, 22)	0.97
Basal FSH, mIU/mL	5.8 (4.7, 7.1)	6.1 (4.7, 7.4)	5.8 (4.7, 7.0)	5.7 (4.7, 6.9)	5.6 (4.6, 7.0)	0.21
FPG, mmol/L	4.9 (4.6, 5.2)	4.7 (4.4, 4.9)	4.9 (4.6, 5.1)	4.9 (4.7, 5.2)	5.3 (4.8, 5.7)	< 0.001
2hPG, mmol/L	6.8 (5.8, 8.2)	5.9 (5.2, 6.8)	6.5 (5.7, 7.7)	7.2 (6.2, 8.5)	8.2 (6.9, 10.2)	< 0.001
FPI, uIU/mL	12.0 (8.1, 17.0)	6.5 (5.2, 7.6)	10.5 (9.5, 11.4)	14.5 (13.0, 15.9)	22.6 (19.7, 27.0)	< 0.001
2hPI, uIU/mL	77.6 (43.5, 131.1)	40.3 (26.9, 59.3)	65.7 (42.8, 94.8)	96.3 (66.0, 134.6)	170.6 (113.6, 241.0)	< 0.001
HbA1c, %	5.5 (5.3, 5.7)	5.4 (5.2, 5.6)	5.4 (5.2, 5.7)	5.5 (5.3, 5.7)	5.6 (5.5, 5.9)	< 0.001
Serum TC, mmol/L	4.6 (4.1, 5.1)	4.5 (4.0, 5.1)	4.6 (4.0, 5.1)	4.6 (4.1, 5.2)	4.6 (4.0, 5.1)	0.55
Serum TG, mmol/L	1.4 (1.0, 2.1)	1.0 (0.7, 1.5)	1.4 (0.9, 1.9)	1.6 (1.1, 2.2)	1.8 (1.3, 2.7)	< 0.001
Serum HDL-C, mmol/L	1.2 (1.0, 1.4)	1.3 (1.1, 1.6)	1.2 (1.1, 1.4)	1.1 (1.0, 1.3)	1.1 (0.9, 1.2)	< 0.001
Serum LDL-C, mmol/L	2.9 (2.5, 3.4)	2.8 (2.4, 3.3)	2.9 (2.4, 3.5)	3.0 (2.6, 3.5)	3.0 (2.6, 3.5)	< 0.01
Duration of infertility, years	3 (2, 5)	3 (2, 4)	3 (2, 5)	3 (2, 5)	4 (2, 5)	0.02
Infertility type						0.07
Primary	654 (71.3%)	195 (76.8%)	150 (70.4%)	167 (71.4%)	142 (65.7%)	
Secondary	263 (28.7%)	59 (23.2%)	63 (29.6%)	67 (28.6%)	74 (34.3%)	
Ovarian stimulation regimen						0.51
GnRH antagonist	762 (83.1%)	211 (83.1%)	178 (83.6%)	191 (81.6%)	182 (84.3%)	
Long GnRHa	145 (15.8%)	38 (15.0%)	32 (15.0%)	41 (17.5%)	34 (15.7%)	
Others^a^	10 (1.1%)	5 (2.0%)	3 (1.4%)	2 (0.9%)	0 (0.0%)	
Insemination technique						0.02
IVF	674 (73.5%)	168 (66.1%)	161 (75.6%)	177 (75.6%)	168 (77.8%)	
ICSI	243 (26.5%)	86 (33.9%)	52 (24.4%)	57 (24.4%)	48 (22.2%)	
Number of transferred embryos						0.09
1	110 (12.0%)	22 (8.6%)	26 (12.2%)	39 (16.7%)	23 (10.6%)	
2	807 (88.0%)	232 (91.3%)	187 (87.8%)	195 (83.3%)	193 (89.4%)	
Timing of embryo transfer						0.03
Day 3	871 (95.0%)	245 (96.5%)	205 (96.2%)	212 (90.6%)	209 (96.8%)	
Day 5	27 (2.9%)	7 (2.8%)	4 (1.9%)	11 (4.7%)	5 (2.3%)	
Day 6	19 (2.1%)	2 (0.8%)	4 (1.9%)	11 (4.7%)	2 (0.9%)	

Numbers are median ± IQR (range), except for percentages

PCOS, polycystic ovary syndrome; IVF, in vitro fertilization; ICSI, intracytoplasmic sperm injection; HOMA2-IR, homeostasis modelsl assessment 2 estimate of insulin resistance; BMI, body mass index; AFC, antral follicle count; FSH, follicle-stimulating hormone; FPG, fasting plasma glucose; 2hPG, 2-h plasma glucose; FPI, fasting plasma insulin; 2hPI, 2-h plasma insulin; HbA1c, glycosylated hemoglobin type A1C; TC, total cholesterol; TG, triglyceride; HDL-C, high-density liptein cholesterol; LDL-C, low-density liptein cholesterol; GnRH, gonadotropin-releasing hormone; GnRHa, gonadotropin-releasing hormone agonist; IQR, interquartile range

### Glucose metabolism indicators and IVF/ICSI outcomes in PCOS women

The effects of glucose metabolism on IVF/ICSI early reproductive outcomes among PCOS women based on the multivariate GLMs are shown in Table 2. After adjustment for age, AFC, and insemination technique, significant monotonic relationships were found across increasing 2hPG, FPI, 2hPI, HbA1c, and HOMA2-IR with decreasing retrieved oocyte count, MII oocyte count, normally fertilized zygote count, normally cleaved embryo count, high-quality embryo count, and blastocyst formation count (All *P* < 0.05). FPG was negatively associated with retrieved oocyte count, MII oocyte count, and blastocyst formation count (All *P* < 0.01). We also observed that increased 2hPI led to a lower chance of live birth (OR: 1.27; 95% CI 1.06, 1.53; *P* = 0.01) after adjustment for age, AFC, and insemination technique (Table 3).

**Table 2:** Relative risk (RR) and 95% CI in early reproductive outcomes associated with glucose metabolism indicators among PCOS women undergoing their first IVF/ICSI cycles based on GLMs (*n* = 917)

Glucose metabolism indicators^a^	Retrieved oocytes, *n*		MII oocytes, *n*		Normally fertilized embryos, *n*		Normally cleaved embryos, *n*		High-quality embryos, *n*		Blastocyst formation, *n*	
	RR (95% CI)	*P*	RR (95% CI)	*P*	RR (95% CI)	*P*	RR (95% CI)	*P*	RR (95% CI)	*P*	RR (95% CI)	*P*
FPG, mmol/L	0.76 (0.64, 0.89)	< 0.001	0.78 (0.66, 0.92)	< 0.01	0.86 (0.70, 1.05)	0.14	0.85 (0.69, 1.04)	0.13	0.87 (0.69, 1.09)	0.24	0.52 (0.32, 0.82)	< 0.01
2hPG, mmol/L	0.90 (0.84, 0.97)	< 0.01	0.91 (0.84, 0.97)	< 0.01	0.90 (0.82, 0.98)	0.02	0.90 (0.82, 0.98)	0.02	0.88 (0.80, 0.98)	0.02	0.68 (0.56, 0.83)	< 0.001
FPI, uIU/mL	0.90 (0.87, 0.94)	< 0.001	0.90 (0.87, 0.94)	< 0.001	0.89 (0.85, 0.93)	< 0.001	0.89 (0.85, 0.93)	< 0.001	0.88 (0.83, 0.92)	< 0.001	0.77 (0.69, 0.84)	< 0.001
2hPI, uIU/mL	0.97 (0.94, 0.99)	< 0.01	0.97 (0.94, 1.00)	0.02	0.94 (0.91, 0.97)	< 0.001	0.94 (0.91, 0.97)	< 0.001	0.93 (0.90, 0.97)	< 0.001	0.87 (0.81, 0.94)	< 0.001
HbA1c, %	0.62 (0.47, 0.81)	< 0.001	0.64 (0.48, 0.85)	< 0.01	0.53 (0.38, 0.76)	< 0.001	0.52 (0.37, 0.74)	< 0.001	0.49 (0.33, 0.74)	< 0.001	0.34 (0.15, 0.72)	< 0.01
HOMA2-IR	0.91 (0.87, 0.95)	< 0.001	0.91 (0.87, 0.96)	< 0.001	0.86 (0.81, 0.91)	< 0.001	0.86 (0.81, 0.91)	< 0.001	0.86 (0.81, 0.92)	< 0.001	0.72 (0.64, 0.82)	< 0.001

Adjusted for age (continuous), AFC (continuous), and insemination technique

^a^Transformed by natural logarithm

**Table 3:** Odds ratio (OR) and 95% CI in pregnancy outcomes associated with glucose metabolism indicators among PCOS women undergoing their first IVF/ICSI cycles based on GLMs (*n* = 917)

Glucose metabolism indicators^a^	Biochemical pregnancy		Clinical pregnancy		Live birth	
	OR (95% CI)	*P*	OR (95% CI)	*P*	OR (95% CI)	*P*
FPG, mmol/L	0.98 (0.35, 2.73)	0.97	0.91 (0.32, 2.57)	0.85	1.04 (0.40, 3.79)	0.75
2hPG, mmol/L	1.35 (0.86, 2.13)	0.19	1.23 (0.78, 1.95)	0.38	1.57 (0.95, 2.61)	0.08
FPI, uIU/mL	0.98 (0.78, 1.24)	0.87	1.00 (0.79, 1.27)	0.98	1.15 (0.89, 1.49)	0.27
2hPI, uIU/mL	1.13 (0.95, 1.33)	0.17	1.15 (0.97, 1.37)	0.10	1.27 (1.06, 1.53)	0.01
HbA1c, %	6.30 (1.06, 40.46)	0.05	4.90 (0.80, 32.29)	0.09	7.79 (1.08, 62.03)	0.05
HOMA2-IR	1.08 (0.81, 1.46)	0.59	1.02 (0.76, 1.38)	0.87	1.26 (0.91, 1.73)	0.16

Adjusted for age (continuous), AFC (continuous), and insemination technique

^a^Transformed by natural logarithm

### Glucose metabolism indicators and adiposity and lipid metabolism indicators in PCOS women

The associations between glucose metabolism indicators and adiposity and lipid metabolism indicators among PCOS women based on the multivariate GLMs are shown in Table 4. After controlling for age, AFC, and duration of infertility, we found that increasing 2hPG, 2hPI, and HbA1c were significantly associated with increasing BMI, serum TC, serum TG, and serum LDL-C (All *P* < 0.01) and decreasing serum HDL-C (All *P* < 0.001). Besides, increasing FPG, FPI, and HOMA2-IR were significantly associated with increasing BMI, serum TG, and serum LDL-C (All *P* < 0.05) and decreasing serum HDL-C (All *P* < 0.001).

**Table 4:** Regression coefficients and 95% CI in adiposity and lipid metabolism indicators associated with glucose metabolism indicators among PCOS women undergoing their first IVF/ICSI cycles based on GLMs (*n* = 917)

Glucose metabolism indicators	BMI, kg/m^2^		Serum TC, mmol/L		Serum TG, mmol/L		Serum HDL-C, mmol/L		Serum LDL-C, mmol/L	
	*β* (95% CI)	*P*	*β* (95% CI)	*P*	*β* (95% CI)	*P*	*β* (95% CI)	*P*	*β* (95% CI)	*P*
FPG, mmol/L	1.32 (1.22, 1.43)	< 0.001	1.02 (0.93, 1.12)	0.66	1.77 (1.33, 2.36)	< 0.001	0.78 (0.70, 0.87)	< 0.001	1.16 (1.02, 1.33)	0.03
2hPG, mmol/L	1.20 (1.16, 1.25)	< 0.001	1.06 (1.02, 1.11)	< 0.01	1.90 (1.68, 2.15)	< 0.001	0.83 (0.79, 0.87)	< 0.001	1.12 (1.06, 1.19)	< 0.001
FPI, uIU/mL	1.16 (1.15, 1.18)	< 0.001	1.01 (0.99, 1.03)	0.26	1.51 (1.42, 1.60)	< 0.001	0.85 (0.83, 0.87)	< 0.001	1.06 (1.02, 1.09)	< 0.001
2hPI, uIU/mL	1.09 (1.07, 1.10)	< 0.001	1.02 (1.01, 1.04)	< 0.01	1.34 (1.28, 1.40)	< 0.001	0.91 (0.89, 0.92)	< 0.001	1.05 (1.02, 1.07)	< 0.001
HbA1c, %	1.78 (1.55, 2.04)	< 0.001	1.33 (1.13, 1.56)	< 0.001	5.17 (3.18, 8.40)	< 0.001	0.65 (0.54, 0.79)	< 0.001	1.55 (1.23, 1.95)	< 0.001
HOMA2-IR	1.20 (1.18, 1.23)	< 0.001	1.02 (1.00, 1.05)	0.09	1.63 (1.51, 1.76)	< 0.001	0.83 (0.81, 0.86)	< 0.001	1.08 (1.04, 1.12)	< 0.001

^a^Adjusted for age (continuous), AFC (continuous), and duration of infertility

^b^Glucose metabolism, adiposity, and lipid metabolism indicators were transformed by natural logarithm

### Adiposity and lipid metabolism indicators and IVF/ICSI outcomes in PCOS women

We also explored the effect of adiposity and lipid metabolism indicators on PCOS women’s IVF/ICSI early reproductive and pregnancy outcomes. As shown in Additional file 1: Table S1, BMI, serum TG, and serum LDL-C were negatively associated with all six IVF/ICSI early reproductive outcomes (All *P* < 0.05). Serum TC was negatively related to high-quality embryo count (*P* < 0.01). Serum HDL-C was positively associated with all six IVF/ICSI early reproductive outcomes (All *P* < 0.01). However, we found no association between adiposity and lipid metabolism indicators and IVF/ICSI pregnancy outcomes in PCOS women (Additional file 1: Table S2).

### Mediation analysis

After adjusting for various confounders and adiposity and lipid metabolism indicators, we found significant adverse effects of FPG on retrieved oocyte count and MII oocyte count (All *P* < 0.05); significant negative effects of 2hPG, 2hPI, and HbA1c on retrieved oocyte count, MII oocyte count, normally fertilized zygote count, normally cleaved embryo count (All *P* < 0.05); and significant adverse effects of FPI and HOMA2-IR on normally cleaved embryo count (All *P* < 0.05) (Table 5). A significant mediating effect by serum TG was observed for the impact of glucose homeostasis and IR on early reproductive outcomes, with 6.0–31.0% proportion mediated. Moreover, we found statistically significant mediation for the effects of glucose homeostasis and IR on early reproductive outcomes by serum TC (6.1–10.8% proportion mediated), serum HDL-C (9.4–43.6% proportion mediated), serum LDL-C (4.2–18.2% proportion mediated), and BMI (26.7–97.7% proportion mediated) (Additional file 1: Tables S3 to S6).

**Table 5:** Mediating effects investigating whether serum TG mediated the associations between glucose metabolism indicators and IVF/ICSI early reproductive outcomes in PCOS women

Mediators	Associations	Total effect (95% CI)	Mediating effect (95% CI)	Estimated proportion of mediation
Serum TG	Retrieved oocyte count and FPG	− 4.08 (− 7.40, − 1.39)***	− 0.26 (− 0.66, − 0.02)*	6.0
	MII oocyte count and FPG	− 3.29 (− 6.36, − 0.86)**	− 0.24 (− 0.57, − 0.02)*	7.1
Serum TG	Retrieved oocyte count and 2hPG	− 1.30 (− 2.39, − 0.39)**	− 0.23 (− 0.49, − 0.01)*	17.8
	MII oocyte count and 2hPG	− 1.13 (− 2.13, − 0.25)**	− 0.22 (− 0.45, − 0.01)*	19.2
	Normally fertilized zygote count and 2hPG	− 0.03 (− 1.61, − 0.09)*	− 0.24 (− 0.45, − 0.05)*	29.1
	Normally cleaved embryo count and 2hPG	− 0.83 (− 1.68, − 0.12)*	− 0.25 (− 0.45, − 0.08)**	31.0
Serum TG	Normally cleaved embryo count and FPI	− 0.97 (− 1.45, − 0.55)***	− 0.11 (− 0.22, − 0.01)*	11.1
Serum TG	Retrieved oocyte count and 2hPI	− 0.43 (− 0.81, − 0.10)*	− 0.09 (− 0.20, − 0.01)*	21.5
	MII oocyte count and 2hPI	− 0.35 (− 0.70, − 0.03)*	− 0.09 (− 0.18, − 0.01)*	25.6
	Normally fertilized zygote count and 2hPI	− 0.54 (− 0.89, − 0.23)***	− 0.09 (− 0.18, 0.00)*	16.1
	Normally cleaved embryo count and 2hPI	− 0.54 (− 0.91, − 0.24)***	− 0.09 (− 0.18, − 0.02)*	17.7
Serum TG	Retrieved oocyte count and HbA1c	− 9.84 (− 20.87, − 2.74)***	− 0.93 (− 2.37, − 0.10)*	9.5
	MII oocyte count and HbA1c	− 7.95 (− 17.33, − 1.92)***	− 0.86 (− 1.93, − 0.08)*	10.9
	Normally fertilized zygote count and HbA1c	− 9.46 (− 20.11, − 2.35)***	− 1.09 (− 2.47, − 0.20)*	11.8
	Normally cleaved embryo count and HbA1c	− 10.17 (− 22.40, − 2.92)***	− 1.17 (− 2.72, − 0.26)**	12.1
Serum TG	Normally cleaved embryo count and HOMA2-IR	− 0.96 (− 1.33, − 0.59)***	− 0.11 (− 0.21, − 0.01)*	10.9

Adjusted for age (continuous), AFC (continuous), and insemination technique

Glucose metabolism indicators and serum TG were transformed by natural logarithm

*P < 0.05

**P < 0.01

***P < 0.001

## Discussion

In this study focused on women with PCOS, we observed significant dose-dependent correlations between glucose metabolism indicators and IVF/ICSI early reproductive outcomes and between glucose metabolism indicators and adiposity and lipid metabolism indicators. Moreover, we observed significant dose-dependent correlations between adiposity and lipid metabolism indicators and IVF/ICSI early reproductive outcomes. Our estimates suggested that serum TG, serum HDL-C, serum LDL-C, and BMI mediate the inverse associations between glucose metabolism indicators and all six IVF/ICSI early reproductive outcomes in PCOS women. We also observed that serum TC mediates the inverse associations between glucose metabolism indicators and high-quality embryo count in PCOS women.

Glucose homeostasis dysregulation, IR, obesity, and dyslipidemia are critical clinical manifestations of PCOS. The metabolisms of glucose and lipid are often intertwined and regulated [34, 35]. Most evidence suggests altered lipid concentrations are secondary to IR, IGT, or diabetes [36]. High-sugar diet-induced diabetes and genetically diabetes (*db/db*) mice can cause obesity and dyslipidemia by either microbiota imbalance or deficiency of leptin receptor [37, 38]. Obesity and dyslipidemia could also contribute to the pathogenesis of IR or glucose homeostasis dysregulation. Evidence from animal studies has proved that obesity and dyslipidemia can cause hyperinsulinemia and IR in high-fat diet (HFD)-induced obese and genetically obese (*ob/ob*) mice [39]. Lipids can influence glucose metabolism via several pathways, including disturbing insulin secretion from pancreatic β-cells [40], apoptosis [41], stress responses (e.g., oxidative stress, endoplasmic reticulum stress, and autophagy) [42], and mitochondrial dysfunction [43]. As an instrumental variable method investigating the causal effects of traits using genetic predictors, MR has been used to determine the causal paths among glucose, insulin, and lipid metabolism markers in humans [24, 25, 44, 45]. The causal roles of adiposity and lipid metabolism markers for diabetes have been elucidated [24]. Several studies found that elevated BMI, TG, and small HDL particle levels might increase the risk of diabetes, while elevated large HDL particle levels might have a protective effect [24, 46]. Recently, Zhu et al. systematically investigated the associations among serum HDL-C, LDL-C, TG, fasting insulin, and HbA1c in European populations and illustrated the bidirectional causality of lipids and glycemic traits [25]. They indicated that elevated serum fasting insulin level was a decisive causal factor of low serum HDL-C and high serum TG levels, which in turn would further increase serum fasting insulin [25]. We found significant positive effects of glucose metabolism indicators on serum TC, serum TG, serum LDL-C, and BMI and negative effects of glucose metabolism indicators on serum HDL-C. These findings also prove the close relationships between glucose metabolism indicators and adiposity and lipid metabolism indicators.

We found negative associations between adiposity and lipid metabolism indicators and IVF/ICSI early reproductive outcomes in PCOS women. Numerous previously published literature can support our findings. Increasing BMI was found to be associated with poorer ovarian response [47, 48], fewer retrieved oocytes [47–49], fewer mature oocytes [49], lower clinical pregnancy rate [16, 17, 50], lower live birth rate [16, 51, 52], and higher risk of miscarriage [48, 51] among PCOS women undergoing IVF/ICSI cycles. High serum TC level was found to be a risk factor for the live birth of IVF/ICSI cycles in PCOS women [52, 53]. The mechanism of obesity and dyslipidemia for PCOS women may be intricate. By promoting the mobilization of free fatty acids, IR is a recognized cause of dyslipidemia in PCOS [54]. Obesity can alter the adipokine distribution in adipocytes, thus, resulting in abnormal activation of pro-inflammatory response, accumulated toxic reactive oxygen species, and aggravated oxidative stress [55]. Inflammation and oxidative stress have been illustrated to cause abnormal oogenesis and folliculogenesis [56]. The damage of obesity to oocytes and embryos may also be related to the alteration of epigenetic reprogramming [56]. Animal studies have elucidated that maternal obesity can cause a remarkable reduction of an essential maternal effect protein named *Stella* (aka *Dppa3/Pgc7*) in oocytes and may disrupt the establishment of pronuclear epigenetic asymmetry and impair embryo development via accumulation of maternal 5-hydroxymethylcytosine (*5-hmc*) modifications and DNA lesions [11].

We also revealed inverse associations between PCOS women’s glucose metabolism indicators and IVF/ICSI early reproductive outcomes after controlling for various confounders and adiposity lipid metabolism indicators. Evidence from previous clinical and animal studies supports our results. A secondary analysis of a multicenter randomized trial illustrated that MetS in PCOS women had a negative impact on IVF cycle stimulation characteristics and clinical outcomes, including higher and longer doses of gonadotropin, lower peak estradiol levels, fewer retrieved oocytes, fewer available embryos, and lower oocyte utilization rate [14]. In another clinical trial, elevated serum insulin, HOMA-IR, and homocysteine levels were negatively associated with oocyte numbers and maturation in PCOS women [19]. The mechanism by which glucose metabolism dysfunction leads to abnormal oogenesis and embryogenesis remains unclear. T2DM was found to increase oocyte mtDNA mutations [57]. Studies also found that IR contributed to apoptosis, oxidative stress, impaired mitochondrial function, abnormal spindles, and misaligned chromosomes of oocytes; thus, leading to fertilization disorders and arrest of embryo development [58]. Moreover, preconception hyperglycemia can reduce the expression of *Tet3* dioxygenase in the oocytes, which is responsible for 5-methylcytosine (*5-mc*) oxidation and DNA demethylation in the zygote. This epigenetic alteration disturbed the subsequent DNA methylation reprogramming of embryos [12]. Our mediation analysis showed that TG, TC, HDL-C, LDL-C, and BMI mediate the associations between glucose metabolism indicators and IVF/ICSI early reproductive outcomes, suggesting that obesity and dyslipidemia may mediate the adverse effect of glucose homeostasis dysregulation and IR on oocyte and embryo quality. As we have discussed above, glucose homeostasis dysregulation and IR have been associated with dyslipidemia and may be the causality of dyslipidemia [25], possibly by increasing mobilization of free fatty acids [54]. Animal and human studies also supported the adverse effect of obesity and dyslipidemia on IVF/ICSI outcomes via inflammation, oxidative stress, and epigenetic mechanisms [49, 55, 57]. However, we must cautiously interpret the mediation analysis’s results because solid assumptions are required to interpret our findings’ causality.

To our knowledge, this is the first study that conducted a mediation analysis to assess the potential role of adiposity and lipid metabolism indicators in the effects of glucose metabolism indicators on IVF/ICSI outcomes in PCOS women. Our findings provide insights into the mechanism of abnormal oogenesis and embryogenesis caused by glucose metabolism dysfunction and show the importance of the dynamic equilibrium of glucose and lipid metabolism in oogenesis and embryogenesis in PCOS. However, some limitations of this study warrant consideration. First, given the retrospective nature of this study, we cannot entirely avoid the bias in data collection. However, the bias in data collection can be eliminated to some extent because the data we studied are objective measures with specific definitions. Second, the database did not fully record information on adjunctive therapies, including diet, exercise, or medication (i.e., metformin) before or during IVF treatment. Future well-designed prospective studies should focus on the impact of adjunctive therapies. Third, multiple hypothesis tests were simultaneously performed to assess the associations between glucose metabolism indicators and adiposity and lipid metabolism indicators. Thus, we cannot entirely rule out the possibility that some of our results are found by chance. Fourth, we only included PCOS women from a reproductive center, thus possibly causing selection bias and limiting the extrapolation of our results to the general PCOS population. However, these findings may have significance for those who seek infertility treatment, which accounts for 40 to 72% of PCOS women [2, 59]. Fifth, although we found that abnormal glucose metabolism is associated with PCOS women’s adverse pregnancy outcomes in this study, we found no mediating role of obesity and dyslipidemia in this relationship. Further mechanism studies are still needed.

## Conclusions

In conclusion, in the present study on 917 PCOS women undergoing their first IVF/ICSI cycles, we found that glucose metabolism indicators were associated with adiposity and lipid metabolism indicators (i.e., increased TG, TC, LDL-C, and BMI and decreased HDL-C), and adiposity and lipid metabolism indicators were inversely correlated with IVF/ICSI early reproductive outcomes (i.e., reduced retrieved oocyte count, MII oocyte count, normally fertilized zygote count, normally cleaved embryo count, high-quality embryo count, or blastocyst formation count). Mediation analyses suggested that obesity and dyslipidemia may mediate a significant proportion of the adverse effect of glucose homeostasis dysregulation and IR on IVF/ICSI early reproductive outcomes. Our findings show the importance of preconception glucose and lipid management in PCOS women.

## Supplementary Information

**Additional file 1: Table S1. **Relative risk (RR) and 95% CI in early reproductive outcomes associated with adiposity and lipid metabolism indicators among PCOS women undergoing their first IVF/ICSI cycles based on GLMs (*n* = 917). **Table S2.** Odds ratio (OR) and 95% CI in pregnancy outcomes associated with adiposity and lipid metabolism indicators among PCOS women undergoing their first IVF/ICSI cycles based on GLMs (*n* = 917). **Table S3.** Mediating effects investigating whether serum TC mediated the associations between glucose metabolism indicators and IVF/ICSI early reproductive outcomes in PCOS women. **Table S4.** Mediating effects investigating whether serum HDL-C mediated the associations between glucose metabolism indicators and IVF/ICSI early reproductive outcomes in PCOS women. **Table S5. **Mediating effects investigating whether serum LDL-C mediated the associations between glucose metabolism indicators and IVF/ICSI early reproductive outcomes in PCOS women. **Table S6.** Mediating effects investigating whether BMI mediated the associations between glucose metabolism indicators and IVF/ICSI early reproductive outcomes in PCOS women.

## Data Availability

The data underlying this article will be shared on reasonable request to the corresponding author.

## Abbreviations

PCOS: Polycystic ovary syndrome
IVF: In vitro fertilization
ICSI: Intracytoplasmic sperm injection
ART: Assisted reproductive technology
BMI: Body mass index
MR: Mendelian randomization
FPG: Fasting plasma glucose
FPI: Fasting plasma insulin
2hPG: 2-Hour plasma glucose
2hPI: 2-Hour plasma insulin
HOMA2-IR: Homeostasis model assessment 2 estimate of insulin resistance
GLM: Generalized linear model
