# Supplementary material for: Adiposity and lipid metabolism indicators mediate the adverse effect of glucose metabolism indicators on oogenesis and embryogenesis in PCOS women undergoing IVF/ICSI cycles
Source: Eur J Med Res. 2023 Jul 3;28:216. doi: 10.1186/s40001-023-01174-8 (PMC10316576; doi:10.1186/s40001-023-01174-8)
Supplement: Supplementary file 1 — Additional file 1: Table S1. Relative risk (RR) and 95% CI in early reproductive outcomes associated with adiposity and lipid metabolism indicators among PCOS women undergoing their first IVF/ICSI cycles based on GLMs (n = 917). Table S2. Odds ratio (OR) and 95% CI in pregnancy outcomes associated with adiposity and lipid metabolism indicators among PCOS women undergoing their first IVF/ICSI cycles based on GLMs (n = 917). Table S3. Mediating effects investigating whether serum TC mediated the associations between glucose metabolism indicators and IVF/ICSI early reproductive outcomes in PCOS women. Table S4. Mediating effects investigating whether serum HDL-C mediated the associations between glucose metabolism indicators and IVF/ICSI early reproductive outcomes in PCOS women. Table S5. Mediating effects investigating whether serum LDL-C mediated the associations between glucose metabolism indicators and IVF/ICSI early reproductive outcomes in PCOS women. Table S6. Mediating effects investigating whether BMI mediated the associations between glucose metabolism indicators and IVF/ICSI early reproductive outcomes in PCOS women. [file 40001_2023_1174_MOESM1_ESM.docx]

| **Table S1.** Relative risk (RR) and 95% CI in early reproductive outcomes associated with adiposity and lipid metabolism indicators among PCOS women undergoing their first IVF/ICSI cycles based on GLMs (n=917)^a^. | | | | | | | | | | | | |
| --- | --- | --- | --- | --- | --- | --- | --- | --- | --- | --- | --- | --- |
| **Adiposity and lipid metabolism indicators^b^** | **Retrieved oocytes, n** | | **MII oocytes, n** | | **Normally fertilized embryos, n** | | **Normally cleaved embryos, n** | | **High-quality embryos, n** | | **Blastocyst formation, n** | |
|  | **RR (95% CI)** | ***P*** | **RR (95% CI)** | ***P*** | **RR (95% CI)** | ***P*** | **RR (95% CI)** | ***P*** | **RR (95% CI)** | ***P*** | **RR (95% CI)** | ***P*** |
| **BMI, kg/m^2^** | 0.68 (0.60, 0.76) | <0.001 | 0.69 (0.69, 0.78) | <0.001 | 0.64 (0.55, 0.75) | <0.001 | 0.64 (0.55, 0.75) | <0.001 | 0.62 (0.52, 0.75) | <0.001 | 0.49 (0.35, 0.69) | <0.001 |
| **Serum TC, mmol/L** | 0.93 (0.83, 1.04) | 0.19 | 0.92 (0.82, 1.03) | 0.14 | 0.90 (0.78, 1.04) | 0.14 | 0.88 (0.76, 1.01) | 0.06 | 0.77 (0.66, 0.91) | <0.01 | 0.82 (0.61, 1.11) | 0.21 |
| **Serum TG, mmol/L** | 0.93 (0.89, 0.96) | <0.001 | 0.92 (0.89, 0.95) | <0.001 | 0.89 (0.85, 0.93) | <0.001 | 0.89 (0.85, 0.93) | <0.001 | 0.90 (0.85, 0.94) | <0.001 | 0.80 (0.73, 0.89) | <0.001 |
| **Serum HDL-C, mmol/L** | 1.13 (1.03, 1.24) | <0.001 | 1.16 (1.05, 1.28) | <0.01 | 1.28 (1.14, 1.44) | <0.001 | 1.28 (1.14, 1.44) | <0.001 | 1.28 (1.11, 1.47) | <0.001 | 1.71 (1.32, 2.21) | <0.001 |
| **Serum LDL-C, mmol/L** | 0.93 (0.86, 1.00) | 0.05 | 0.92 (0.85, 0.99) | 0.04 | 0.89 (0.81, 0.98) | 0.02 | 0.87 (0.79, 0.96) | <0.01 | 0.81 (0.73, 0.91) | <0.001 | 0.76 (0.62, 0.94) | 0.01 |
| ^a^ Adjusted for age (continuous), AFC (continuous), and insemination technique.  ^b^ Transformed by natural logarithm. | | | | | | | | | | | | |

| **Table S2.** Odds ratio (OR) and 95% CI in pregnancy outcomes associated with adiposity and lipid metabolism indicators among PCOS women undergoing their first IVF/ICSI cycles based on GLMs (n=917)^a^. | | | | | | |
| --- | --- | --- | --- | --- | --- | --- |
| **Adiposity and lipid metabolism indicators^b^** | **Biochemical pregnancy** | | **Clinical pregnancy** | | **Live birth** | |
|  | **OR (95% CI)** | ***P*** | **OR (95% CI)** | ***P*** | **OR (95% CI)** | ***P*** |
| **BMI, kg/m^2^** | 1.69 (0.76, 3.78) | 0.20 | 1.72 (0.76, 3.91) | 0.20 | 2.21 (0.92, 5.38) | 0.08 |
| **Serum TC, mmol/L** | 0.83 (0.41, 1.69) | 0.62 | 0.95 (0.46, 1.94) | 0.88 | 0.98 (0.45, 2.12) | 0.96 |
| **Serum TG, mmol/L** | 1.05 (0.83, 1.32) | 0.69 | 1.25 (0.99, 1.58) | 0.06 | 1.07 (0.83, 1.37) | 0.59 |
| **Serum HDL-C, mmol/L** | 0.77 (0.42, 1.41) | 0.39 | 0.83 (0.45, 1.55) | 0.56 | 0.88 (0.45, 1.71) | 0.70 |
| **Serum LDL-C, mmol/L** | 0.98 (0.60, 1.61) | 0.95 | 0.98 (0.59, 1.62) | 0.93 | 1.19 (0.70, 2.04) | 0.52 |
| ^a^ Adjusted for age (continuous), AFC (continuous), and insemination technique.  ^b^ Transformed by natural logarithm. | | | | | | |

| **Table S3.** Mediating effects investigating whether serum TC mediated the associations between glucose metabolism indicators and IVF/ICSI early reproductive outcomes in PCOS women^a,b^. | | | | |
| --- | --- | --- | --- | --- |
| **Mediators** | **Associations** | **Total effect (95% CI)** | **Mediating effect (95% CI)** | **Estimated proportion of mediation** |
| Serum TC | High-quality embryo count and 2hPG | **–**0.72 (**–**1.42, **–**0.09)* | **–**0.08 (**–**0.18, **–**0.01)** | 10.8 |
| Serum TC | High-quality embryo count and 2hPI | **–**0.43 (**–**0.73, **–**0.16)** | **–**0.03 (**–**0.06, 0.00)* | 6.1 |
| Serum TC | High-quality embryo count and HbA1c | **–**8.72 (**–**20.29, **–**2.02)*** | **–**0.80 (**–**1.98, **–**0.15)** | 9.4 |
| ^a^ Adjusted for age (continuous), AFC (continuous), and insemination technique.  ^b^ Glucose metabolism indicators and serum TC were transformed by natural logarithm. * P<0.05. ** P<0.01. *** P<0.001. | | | | |

| **Table S4.** Mediating effects investigating whether serum HDL-C mediated the associations between glucose metabolism indicators and IVF/ICSI early reproductive outcomes in PCOS women^a,b^. | | | | |
| --- | --- | --- | --- | --- |
| **Mediators** | **Associations** | **Total effect (95% CI)** | **Mediating effect (95% CI)** | **Estimated proportion of mediation** |
| Serum HDL-C | Retrieved oocyte count and FPG | –4.02 (–7.20, –1.26)*** | –0.39 (–0.83, –0.03)* | 9.8 |
|  | MII oocyte count and FPG | –3.28 (–6.27, –0.85)*** | –0.46 (–0.92, –0.15)*** | 14.3 |
|  | Blastocyst formation count and FPG | –2.04 (–5.15, –0.29)** | –0.35 (–0.75, –0.11)*** | 18.2 |
| Serum HDL-C | Retrieved oocyte count and 2hPG | –1.27 (–2.24, –0.34)** | –0.23 (–0.47, 0.00)* | 18.8 |
|  | MII oocyte count and 2hPG | –1.14 (–2.12, –0.28)** | –0.29 (–0.53, –0.09)** | 25.6 |
|  | Normally fertilized zygote count and 2hPG | –0.80 (–1.67, –0.08)* | –0.33 (–0.55, –0.15)** | 41.1 |
|  | Normally cleaved embryo count and 2hPG | –0.81 (–1.64, –0.12)* | –0.33 (–0.52, –0.15)*** | 41.6 |
|  | High-quality embryo count and 2hPG | –0.72 (–1.45, –0.10)* | –0.24 (–0.42, –0.08)*** | 33.0 |
|  | Blastocyst formation count and 2hPG | –0.94 (–1.80, –0.29)*** | –0.18 (–0.34, –0.06)*** | 20.0 |
| Serum HDL-C | Normally fertilized zygote count and FPI | –1.00 (–1.50, –0.56)*** | –0.20 (–0.38, –0.03)* | 19.9 |
|  | Normally cleaved embryo count and FPI | –0.96 (–1.44, –0.55)*** | –0.20 (–0.37, –0.03)* | 21.1 |
| Serum HDL-C | MII oocyte count and 2hPI | –0.35 (–0.69, –0.04)* | –0.15 (–0.27, –0.04)** | 43.6 |
|  | Normally fertilized zygote count and 2hPI | –0.54 (–0.92, –0.23)*** | –0.16 (–0.28, –0.06)** | 30.2 |
|  | Normally cleaved embryo count and 2hPI | –0.54 (–0.91, –0.24)*** | –0.16 (–0.27, –0.06)*** | 30.6 |
|  | High-quality embryo count and 2hPI | –0.43 (–0.74, –0.16)*** | –0.12 (–0.21, –0.03)* | 27.3 |
|  | Blastocyst formation count and 2hPI | –0.32 (–0.61, –0.11)*** | –0.09 (–0.16, –0.03)** | 27.6 |
| Serum HDL-C | Retrieved oocyte count and HbA1c | –9.66 (–19.87, –2.44)*** | –0.90 (–2.01, –0.08)* | 9.4 |
|  | MII oocyte count and HbA1c | –7.96 (–17.03, –1.88)*** | –1.03 (–2.17, –0.30)*** | 13.4 |
|  | Normally fertilized zygote count and HbA1c | –9.45 (–21.43, –2.35)*** | –1.44 (–2.98, –0.52)** | 15.7 |
|  | Normally cleaved embryo count and HbA1c | –10.08 (–22.24, –2.88)*** | –1.46 (–2.96, –0.54)*** | 15.2 |
|  | High-quality embryo count and HbA1c | –8.83 (–20.93, –2.08)*** | –1.14 (–2.72, –0.30)*** | 13.4 |
|  | Blastocyst formation count and HbA1c | –7.63 (–25.67, –0.68)** | –1.29 (–3.67, –0.26)*** | 19.0 |
| Serum HDL-C | MII oocyte count and HOMA2-IR | –0.90 (–1.37, –0.45)*** | –0.19 (–0.39, –0.01)* | 20.8 |
|  | Normally fertilized zygote count and HOMA2-IR | –0.97 (–1.36, –0.59)*** | –0.20 (–0.35, –0.05)** | 20.4 |
|  | Normally cleaved embryo count and HOMA2-IR | –0.96 (–1.33, –0.59)*** | –0.19 (–0.35, –0.05)* | 20.6 |
|  | High-quality embryo count and HOMA2-IR | –0.72 (–1.05, –0.40)*** | –0.14 (–0.27, –0.01)* | 19.8 |
|  | Blastocyst formation count and HOMA2-IR | –0.45 (–0.63, –0.63)*** | –0.08 (–0.15, –0.01)* | 17.0 |
| ^a^ Adjusted for age (continuous), AFC (continuous), and insemination technique.  ^b^ Glucose metabolism indicators and serum HDL-C were transformed by natural logarithm. * P<0.05. ** P<0.01. *** P<0.001. | | | | |

| **Table S5.** Mediating effects investigating whether serum LDL-C mediated the associations between glucose metabolism indicators and IVF/ICSI early reproductive outcomes in PCOS women^a,b^. | | | | |
| --- | --- | --- | --- | --- |
| **Mediators** | **Associations** | **Total effect (95% CI)** | **Mediating effect (95% CI)** | **Estimated proportion of mediation** |
| Serum LDL-C | Normally cleaved embryo count and 2hPG | –0.81 (–1.65, –0.13)* | –0.10 (–0.22, –0.02)* | 12.4 |
|  | High-quality embryo count and 2hPG | –0.72 (–1.44, –0.09)* | –0.13 (–0.26, –0.04)*** | 18.2 |
| Serum LDL-C | Normally cleaved embryo count and FPI | –0.96 (–1.44, –0.56)*** | –0.04 (–0.10, 0.00)* | 4.2 |
|  | High-quality embryo count and FPI | –0.82 (–1.23, –0.44)*** | –0.06 (–0.12, –0.01)** | 6.8 |
| Serum LDL-C | Normally cleaved embryo count and 2hPI | –0.54 (–0.90, –0.24)*** | –0.04 (–0.08, 0.00)* | 6.6 |
|  | High-quality embryo count and 2hPI | –0.43 (–0.73, –0.16)** | –0.05 (–0.10, –0.01)*** | 11.2 |
| Serum LDL-C | High-quality embryo count and HbA1c | –10.13 (–22.31, –2.98)*** | –0.72 (–1.86, –0.07)* | 7.3 |
|  | Blastocyst formation count and HbA1c | –8.79 (–20.54, –2.06)*** | –1.01 (–2.42, –0.27)** | 12.0 |
| Serum LDL-C | Normally cleaved embryo count and HOMA2-IR | –0.96 (–1.32, –0.59)*** | –0.05 (–0.11, 0.00)* | 5.0 |
|  | High-quality embryo count and HOMA2-IR | –0.72 (–1.03, –0.40)*** | –0.07 (–0.13, –0.02)** | 9.2 |
| ^a^ Adjusted for age (continuous), AFC (continuous), and insemination technique.  ^b^ Glucose metabolism indicators and serum LDL-C were transformed by natural logarithm. * P<0.05. ** P<0.01. *** P<0.001. | | | | |

| **Table S6.** Mediating effects investigating whether BMI mediated the associations between glucose metabolism indicators and IVF/ICSI early reproductive outcomes in PCOS women^a,b^. | | | | |
| --- | --- | --- | --- | --- |
| **Mediators** | **Associations** | **Total effect (95% CI)** | **Mediating effect (95% CI)** | **Estimated proportion of mediation** |
| BMI | Retrieved oocyte count and FPG | –4.02 (–7.26, –1.28)*** | –1.45 (–2.30, –0.80)*** | 37.1 |
|  | MII oocyte count and FPG | –3.27 (–6.35, –0.84)** | –1.26 (–1.98, –0.68)*** | 38.8 |
|  | Blastocyst formation count and FPG | –2.11 (–5.49, –0.27)** | –0.53 (–1.13, –0.17)*** | 27.0 |
| BMI | Retrieved oocyte count and 2hPG | –1.26 (–2.25, –0.33)** | –0.85 (–1.24, –0.52)*** | 68.5 |
|  | MII oocyte count and 2hPG | –1.13 (–2.15, –0.25)** | –0.75 (–1.10, –0.45)*** | 66.4 |
|  | Normally fertilized zygote count and 2hPG | –0.80 (–1.66, –0.08)* | –0.60 (–0.90, –0.34)*** | 77.6 |
|  | Normally cleaved embryo count and 2hPG | –0.81 (–1.63, –0.12)* | –0.60 (–0.90, –0.35)*** | 72.9 |
|  | High-quality embryo count and 2hPG | –0.73 (–1.47, –0.10)* | –0.47 (–0.75, –0.25)*** | 64.1 |
|  | Blastocyst formation count and 2hPG | –0.95 (–1.84, –0.32)*** | –0.25 (–0.46, –0.07)** | 26.7 |
| BMI | Retrieved oocyte count and FPI | –1.28 (–1.79, –0.74)*** | –0.57 (–0.89, –0.28)*** | 44.5 |
|  | MII oocyte count and FPI | –1.23 (–1.77, –0.72)*** | –0.48 (–0.76, –0.22)*** | 38.9 |
|  | Normally fertilized zygote count and FPI | –1.00 (–1.50, –0.57)*** | –0.38 (–0.62, –0.15)** | 38.7 |
|  | Normally cleaved embryo count and FPI | –0.96 (–1.43, –0.55)*** | –0.39 (–0.63, –0.16)*** | 40.1 |
|  | High-quality embryo count and FPI | –0.83 (–1.24, –0.45)*** | –0.29 (–0.52, –0.08)** | 35.3 |
| BMI | Retrieved oocyte count and 2hPI | –0.42 (–0.77, –0.08)* | –0.38 (–0.55, –0.24)*** | 92.2 |
|  | MII oocyte count and 2hPI | –0.35 (–0.69, –0.03)* | –0.34 (–0.49, –0.21)*** | 97.7 |
|  | Normally fertilized zygote count and 2hPI | –0.54 (–0.93, –0.23)*** | –0.26 (–0.39, –0.13)*** | 48.7 |
|  | Normally cleaved embryo count and 2hPI | –0.54 (–0.90, –0.24)*** | –0.25 (–0.40, –0.14)*** | 47.6 |
|  | High-quality embryo count and 2hPI | –0.43 (–0.76, –0.16)** | –0.20 (–0.33, –0.10)*** | 47.1 |
|  | Blastocyst formation count and 2hPI | –0.32 (–0.63, –0.11)*** | –0.10 (–0.19, –0.03)** | 31.9 |
| BMI | Retrieved oocyte count and HbA1c | –9.65 (–20.03, –2.70)*** | –4.03 (–7.10, –2.09)*** | 44.0 |
|  | MII oocyte count and HbA1c | –7.93 (–16.89, –1.83)*** | –3.44 (–5.80, –1.74)*** | 45.3 |
|  | Normally fertilized zygote count and HbA1c | –9.44 (–20.60, –2.44)*** | –3.30 (–5.81, –1.45)*** | 37.1 |
|  | Normally cleaved embryo count and HbA1c | –10.12 (–22.62, –2.77)*** | –3.36 (–6.40, –1.53)*** | 34.9 |
|  | High-quality embryo count and HbA1c | –8.87 (–20.57, –2.03)*** | –2.81 (–5.66, –5.66)*** | 33.1 |
|  | Blastocyst formation count and HbA1c | –7.73 (–26.74, –0.57)** | –2.31 (–6.40, –0.49)** | 33.7 |
| BMI | Retrieved oocyte count and HOMA2-IR | –1.01 (–1.49, –0.52)*** | –0.69 (–0.99, –0.40)*** | 67.1 |
|  | MII oocyte count and HOMA2-IR | –0.90 (–1.37, –0.41)*** | –0.61 (–0.89, –0.35)*** | 67.6 |
|  | Normally fertilized zygote count and HOMA2-IR | –0.97 (–1.35, –0.59)*** | –0.39 (–0.62, –0.16)** | 39.4 |
|  | Normally cleaved embryo count and HOMA2-IR | –0.96 (–1.32, –0.59)*** | –0.38 (–0.60, –0.17)*** | 39.4 |
|  | High-quality embryo count and HOMA2-IR | –0.72 (–1.04, –0.40)*** | –0.31 (–0.52, –0.12)** | 42.4 |
| ^a^ Adjusted for age (continuous), AFC (continuous), and insemination technique.  ^b^ Glucose metabolism indicators and BMI were transformed by natural logarithm. * P<0.05. ** P<0.01. *** P<0.001. | | | | |
